# Pulmonary Apoplexy

**Published:** 1829-04-01

**Authors:** 


### XXVI.

### Case of Pulmonary Apoplexy.

Such a disease is not to be found in our systems of nosology—but it is to be seen in practice—and the name is, we believe, just as proper as cerebral apoplexy. We know that, in this last disease, there is not always a rupture of vessels, but a complete engorgement of the vascular system of the brain, by which the functions of the organ are more speedily annihilated than when an artery gives way, a quantity of blood extravasated, and a portion of the brain torn up. It is so with the lungs. In some way which we cannot explain, the organ becomes suddenly gorged with blood, and the patient is actually and literally choaked, in consequence of effusion of blood into the air-cells of the lungs. The following case will illustrate these observations.

M. Del——, aged 29 years, of apparently good constitution, had been, for some months, closely confined to sedentary avocations, and was subject, for some years previously, to stomach-complaints, for which he was in the habit of applying leeches to the anus. On the 31st August, 1828, this gentleman found himself affected with one of his stomach attacks, and confined himself to an abstemious regimen for its removal; but not getting better, he sent for M. Lefranc, a physician, who visited him on the 2d of September. Dr. L. found the patient sitting by the fire, with pallid face, turgid and lachrymose eyes, and complaining of a fixed pain directly under the sternum, accompanied by a sense of heat there, and some difficulty of breathing. Whilst the Doctor was asking him some questions, the patient suddenly exclaimed that the "pain was gone." His face became of a violet hue, he fell to the ground, some frothy saliva issued from his mouth, and he instantly expired.

*Dissection.* The vessels of the head were more than usually exsanguious. The lungs, excepting a small portion at their summit, were of a violet colour, and so gorged with blood as to resemble liver. There was no other disease in the chest. In the stomach and bowels there were marks of derangement, especially in the mucous membrane about the pylorus, and in the small intestines.—*Revue Medicale.*

The above is a striking example of pulmonary apoplexy—and when we reflect on the number of people who die suddenly, without any subsequent examination, we may fairly presume that the disease is not of extremely rare occurrence. We may lay it down as a rule, indeed, that in all, or almost all, *very sudden* deaths, the cause will be found in the heart or lungs, rather than in the brain or spinal marrow. The heart is most frequently the seat of disease in such rapid terminations of life. The most fatal apoplexies (cerebral) require some hours to extinguish the functions of the heart and lungs. What is the nature of these rapid accumulations of blood? The word "determination" is evidently absurd. The blood cannot be *determined* to any particular organ, except by causes acting in the organ itself. "Attraction" of blood would be more proper than "determination."

---

